# Interosseous wiring for fragmented proximal phalangeal fractures

**DOI:** 10.1080/23320885.2022.2039668

**Published:** 2022-02-21

**Authors:** Hidetoshi Teraura, Hideki Sakanaka, Hiroyuki Gotani

**Affiliations:** a Department of Orthopaedic Surgery, Higashisumiyoshi Morimoto Hospital, Osaka city, Osaka, Japan; b Department of Orthopaedic Surgery, Seikeikai Hospital, Sakai city, Osaka, Japan; c Hand and Trauma Microsurgery Center, Osaka Ekisaikai Hospital of Japan Seafarers Relief Association, Osaka city, Osaka, Japan

**Keywords:** Proximal phalangeal fractures, extensor tendon adhesion, joint contracture, interosseous wiring

## Abstract

Fragmented proximal phalangeal fractures are difficult to treat. Fixation with plate and screws often lead to contractures and extensor tendon adhesions. Interosseous wiring could prevent those complications by repairing the periosteum and avoiding direct contact between implants and extensor tendon, while a good total active motion can be achieved.

## Introduction

Proximal phalanx fractures are traumas encountered in regular medical treatment and occur in approximately 1.4% of upper limb fractures aged 18 years and older [1]. The treatment goals for extra-articular fractures of the proximal phalanges of the fingers are to achieve bone healing and functional recovery. These require anatomical reduction, stable fixation, and appropriate postoperative therapy to prevent joint contractures and tendon adhesions. However, in fractures involving multiple fragments, sequelae due to contractures and extensor tendon adhesions persist in some cases, which render the treatment challenging.

In our department, we use conservative therapy if proper alignment can be achieved by manual reduction and the reduced position can be maintained with immobilization . However, in case the alignment achieved by manual reduction cannot be maintained with immobilization, we use percutaneous pinning or screw fixation *via* a small incision. Percutaneous pinning by Kirschner wire intramedullary fixation, which is less invasive, is the first choice for the surgical treatment of extra-articular fractures of the proximal phalanges of the fingers. However, proximal phalangeal fractures involving more than three unstable fragments may render it challenging to obtain optimum fixation by only percutaneous pinning or screw fixation. Although plate and screw osteosynthesis is common surgery, the use of low-profile plates can also result in adhesions between the plate and the extensor tendons [2]. When early range of motion (ROM) training is anticipated to be challenging, we perform interosseous wiring (IOW). This study aimed to investigate the treatment outcomes, advantage, and complications of IOW in proximal phalangeal fractures involving more than three fragments.

## Materials and methods

Ethical approval was obtained from the institutional review board and informed consent was obtained from all participants included in the study. We conducted surgery on patients for whom conservative therapy was unsuccessful. Manual reduction was attempted under local anesthesia. If closed reduction was successful and proper alignment was achieved, percutaneous pinning or screw fixation *via* a small incision was performed. IOW was performed only if closed reduction could not be achieved.

**Figure 1.**
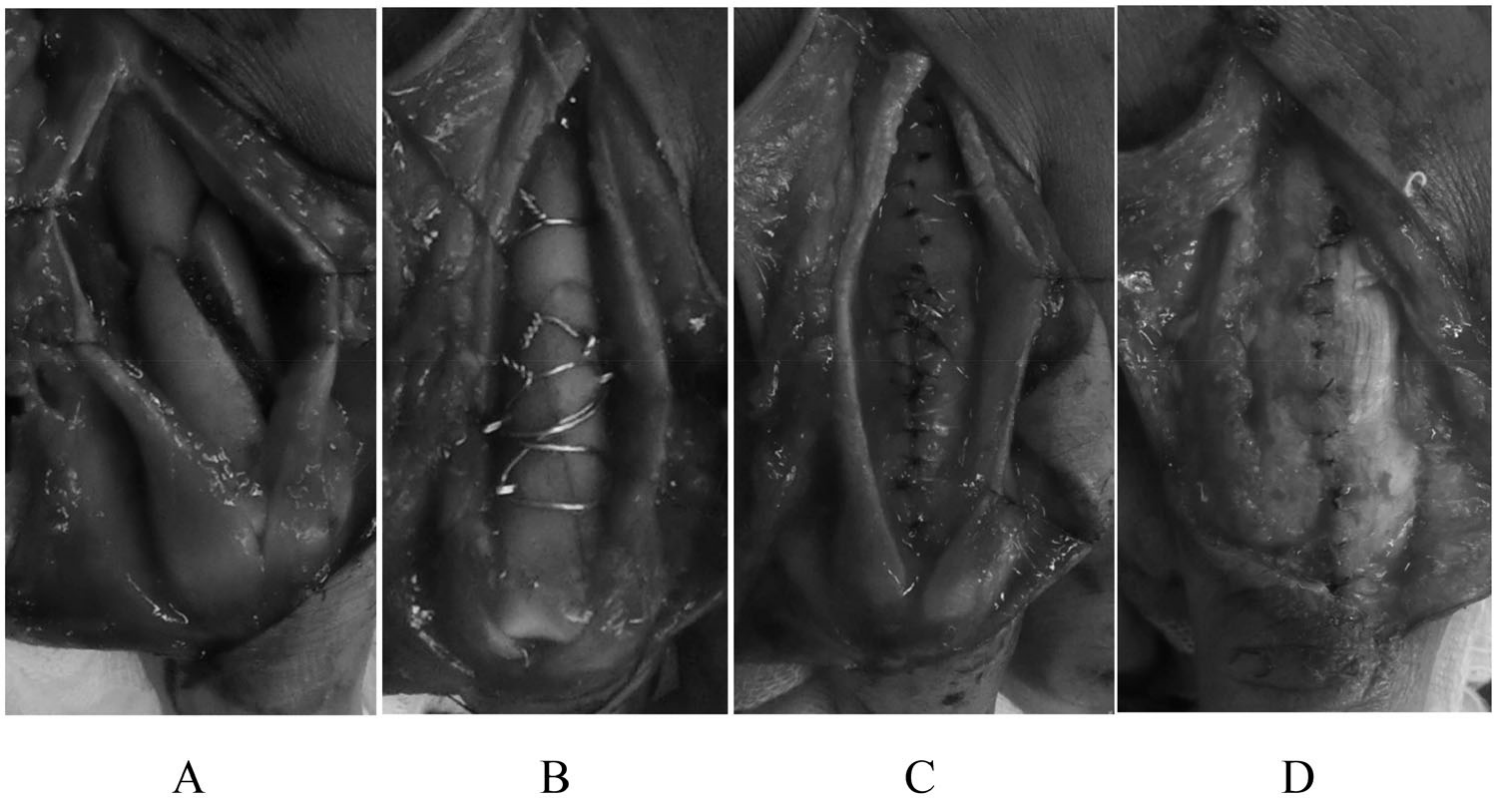
(A) After longitudinally splitting the extensor tendon, the periosteum was carefully detached to expose the fracture site. (B) The bone fragments were individually reduced and fixed with 0.7 mm or 1.0 mm Kirschner wires and 26-gauge or 28-gauge stainless wires. Tension band wiring and circular wiring were used based on the fracture type. (C) The periosteum was repaired using 5-0 nylon, avoiding direct contact between the implant and the extensor tendon to prevent extensor tendon adhesions. (D) The longitudinally split extensor tendon was carefully sutured and repaired using 5-0 nylon, maintaining the PIP joint in flexion.

Five patients with proximal phalangeal fractures treated with IOW between October 2011 and June 2018 were included, with a postoperative observation period of ≥6 months. The surgery was performed by a single surgeon (level 3. Specialist – experienced [3]) under general anesthesia or supraclavicular block. Zig-zag incisions were made on the dorsal skin, depending on the extent of the fracture. After the extensor tendon was longitudinally split, the periosteum was carefully detached to expose the fracture site (Figure 1(A)). Bone fragments were then reduced one by one and fixed using 0.7 mm or 1.0 mm Kirschner wires and 26-gauge (0.405 mm diameter, American wire gauge) or 28-gauge (0.321 mm diameter) stainless steel wires. Tension band wiring and circular wiring were used according to the fracture type (Figure 1(B)). The periosteum was repaired using 5-0 nylon, avoiding direct contact of the implant and extensor tendon to prevent extensor tendon adhesions (Figure 1(C)). The split extensor tendon was carefully sutured and repaired using 5-0 nylon while the proximal interphalangeal (PIP) joint was held in a flexed position (Figure 1(D)). After the surgery, buddy taping was applied to the injured and adjacent fingers, and dorsal fixation was performed using Alfence splints (Alcare Co., Ltd., Tokyo, Japan), maintaining the metacarpophalangeal (MP) joint in a 70° flexion position (Burkhalter fixation). The PIP and distal interphalangeal (DIP) joints were left free to move without fixation, and ROM exercises began immediately after surgery. Flexion contractures can occur even when the PIP joints are left free; therefore, patients were instructed to perform exercises to extend the PIP joints regularly. Three weeks after surgery, the splint was removed, and ROM exercises for the MP joints were initiated.

The following parameters were examined during postoperative assessments: pain, bone healing status, presence or absence of malunion, and postoperative complications, including infection, complex regional pain syndrome (CRPS) type one, re-displacement of the fractured bone and implant breakage, active extension angles of the PIP joint (extension lag), total active motion (TAM), and %TAM. Bone healing was assumed to have occurred if the physical examination no longer revealed tenderness at the fracture site, and X-rays confirmed that continuity of cortical bone had been achieved. A finger goniometer was used to measure the ROM. Clinical assessments were performed using the American Society for Surgery of the Hand (ASSH) criteria [4].

## Results

### Patient characteristics

Patient data and postoperative assessment details are presented in Table 1. Three men and two women with a mean age of 34 (range, 17–68) years were included in the study. A closed fracture occurred in four cases and an open fracture in one case. The delay between injury and surgery was 5 (range, 0–12) days. An average of 3.8 (range, 3–6) bone fragments was recorded. Mean follow-up was 14.8 (range, 8–22) months.

**Table 1.** Individual patient data and postoperative evaluation.

Patient	Sex	Age (years)	Fracture site	Cause of injury	Number of fragments	Follow-up period (months)	PIP joint extension lag (°)	TAM (°)	%TAM (%)	Clinical evaluation
1	M	17	Left little	Sports injuries	3	16	10	245	94	Excellent
2	M	22	Right ring	Traffic accident	3	10	0	260	100	Excellent
3	F	68	Right ring	Twisting accident	3	22	5	230	92	Excellent
4	F	20	Right middle	Sports injuries	4	8	0	240	98	Excellent
5	M	42	Right middle	Occupational accident	6	18	0	210	86	Excellent

TAM: total active motion; PIP: proximal interphalangeal.

### Treatment outcomes

No patient complained of pain. Bone healing was achieved in all cases, and the mean healing time was 13.6 (range, 11–19) weeks. No rotational or flexion/extension deformities were observed. No postoperative complications, such as infection, CRPS type one, re-displacement of the fractured bone, and implant breakage, were reported. The mean extension lag of the PIP joint was 3° (range, 0°–10°), and an extension lag was observed in two cases, one with 5° and another with 10°. The mean TAM was 237° (range, 210°–260°), mean %TAM was 94% (range, 86–100), and the outcome of all five cases was determined to be excellent based on the ASSH criteria (Table 1). The implants were removed after a mean postoperative period of 29 (range, 22–36) weeks in four of the five patients. The remaining patient did not wish for the implant to be removed and was thus left in place. Tenolysis of extensor tendon was not performed during implant removal in any of these cases, and the TAM before and after implant removal was similar.

### Case

**Figure 2.**
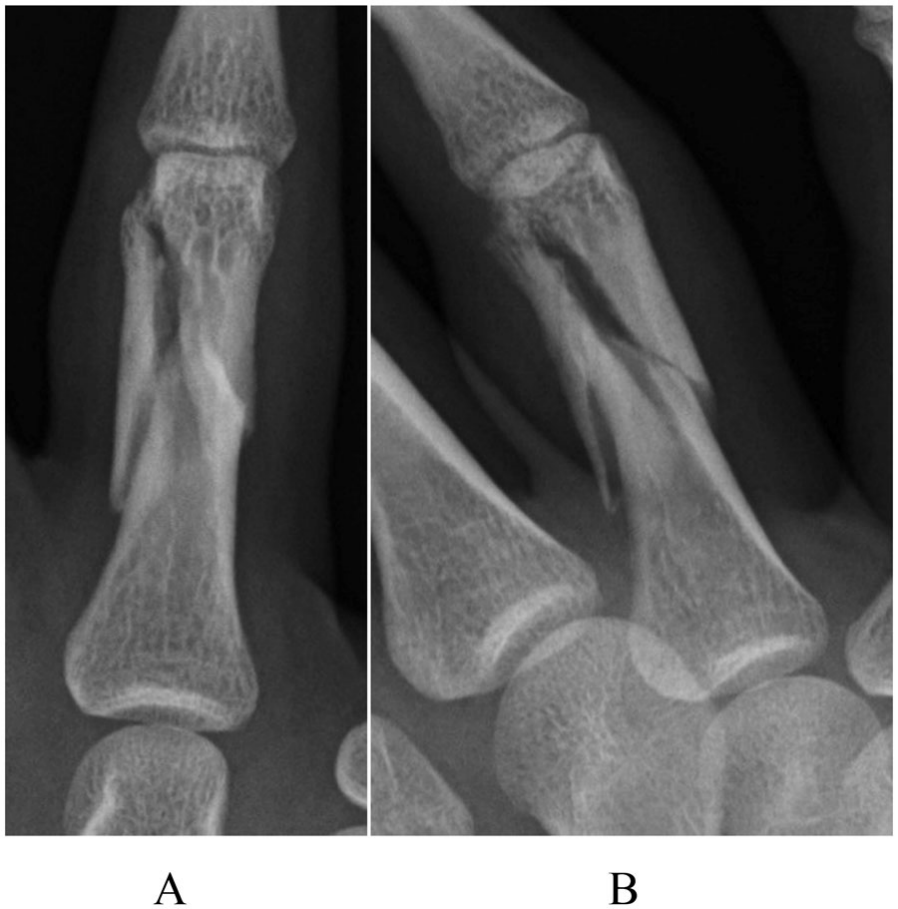
A preoperative radiograph of case 2, showing a displaced three-fragment fracture: (A) antero-posterior view and (B) oblique view.

**Figure 3.**
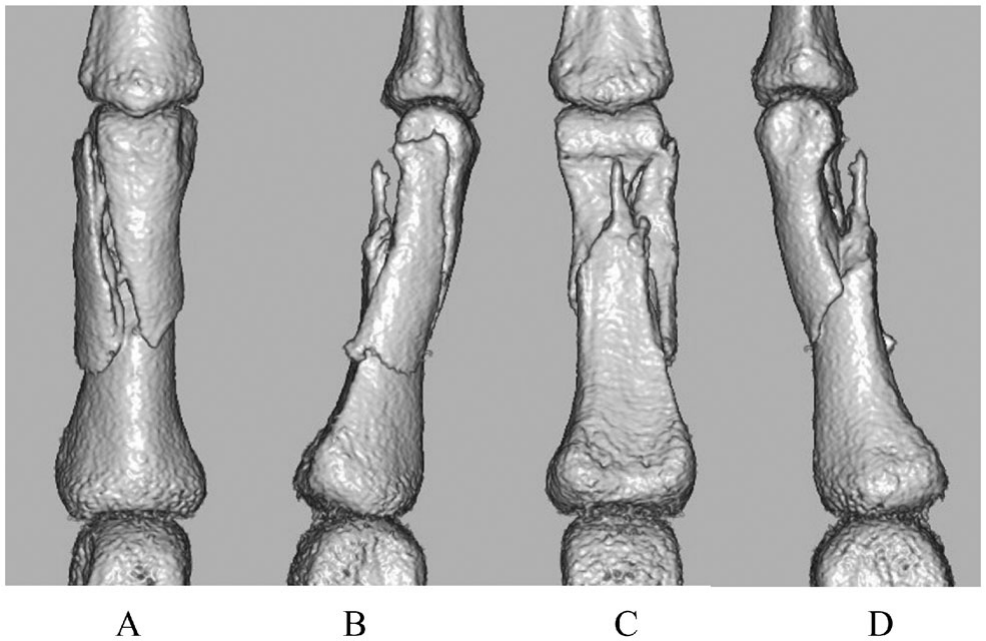
Preoperative three-dimensional computed tomography: (A) dorsal side, (B) radial side, (C) volar side, and (D) ulnar side.

**Figure 4.**
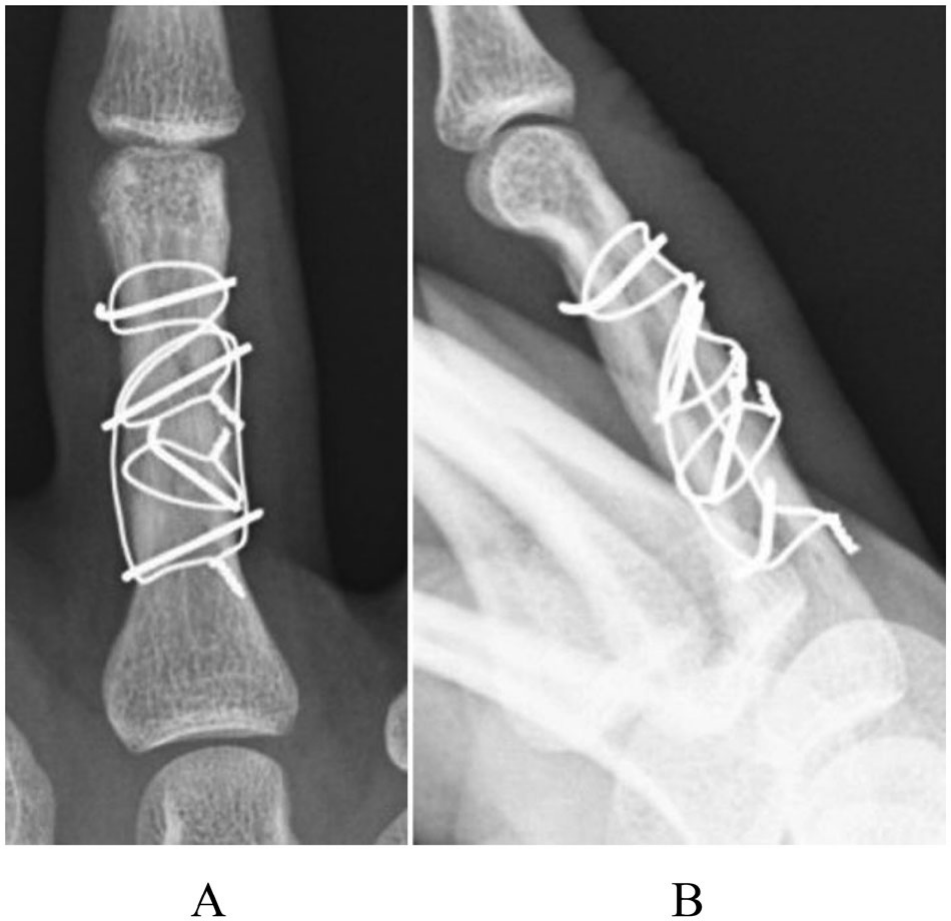
A postoperative radiograph of case 2 after open reduction and internal fixation with IOW, showing proper reduction: (A) antero-posterior view and (B) later al view.

**Figure 5.**
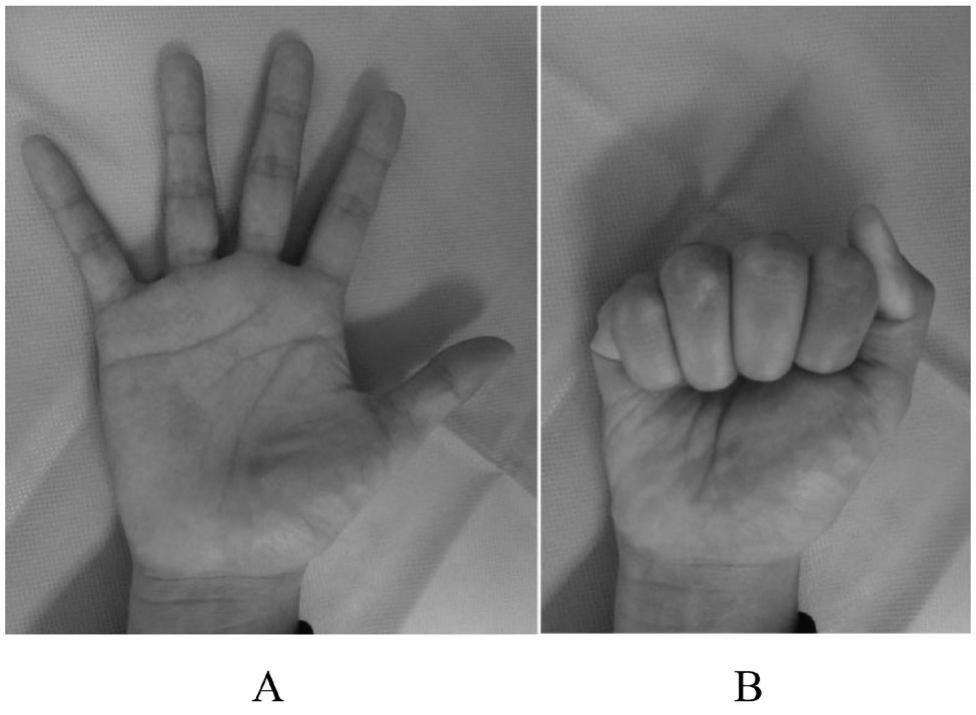
Functional evaluation of case 2, eight weeks after the surgery, showing the ROM of the finger: (A) active extension and (B) active flexion.

**Figure 6.**
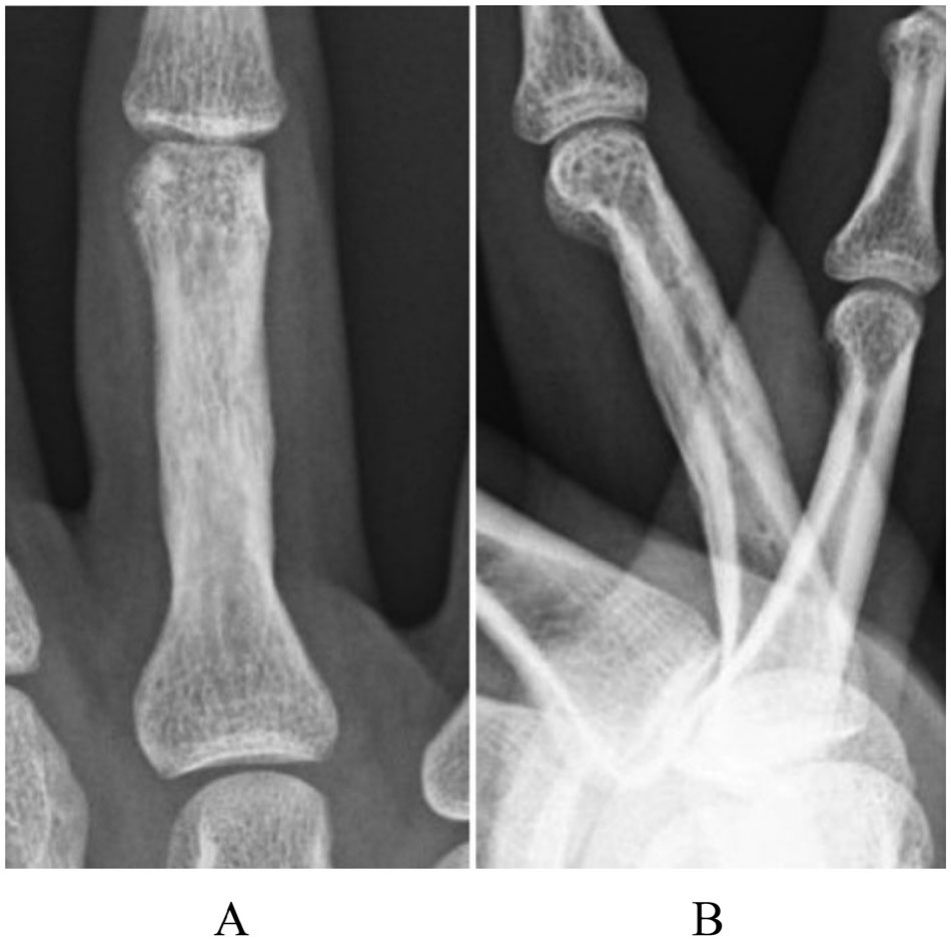
A 10-month postoperative radiograph of case 2, showing complete union without reduction loss: (A) antero-posterior view and (B) later al view.

The patient was a 22-year-old man who was injured following a slip and fall while riding a 400-cc motorcycle. The patient visited a nearby clinic and was referred to our department. Swelling and tenderness were observed at the proximal phalangeal area of the right ring finger. Radiography revealed a three-part fracture from the neck to the shaft of the proximal phalanx of the right ring finger (Figure 2), and small bone fragments were partially seen on three-dimensional computed tomography (Figure 3). Surgery was performed 4 days after the injury, and fracture reduction was confirmed based on anterior and lateral views on a postoperative radiograph (Figure 4). Finger ROM exercises were initiated from the first day after surgery, and a TAM of 260° and a %TAM of 100% were achieved at 8 weeks postoperatively (Figure 5). Bone healing was achieved (Figure 6), and no pain or rotation deformity of the ring finger was reported at the final assessment. Ten months after surgery, at the final evaluation, the outcome was assessed as excellent based on the ASSH criteria.

## Discussion

In the present study, we assessed the clinical outcome of 5 patients with a comminuted fracture of the proximal phalanx (at least three fragments) treated with open reduction and interosseous wiring. We observed successful bone healing and excellent outcomes in all patients and no postoperative complications or deformities. Fractures of the proximal phalanx of the fingers are often seen in daily clinical practice; however, if not treated properly, they might lead to tendon adhesions which cause limited ROM. For a good clinical outcome, the bone fracture must heal properly while retaining as much function as possible. For bone healing, accurate reduction and proper fixation are necessary. For functional recovery, joint contractures and tendon adhesions must be prevented with adequate postoperative therapy combined with proper immobilization [5].

There are various types of fractures of the proximal phalanx of the fingers, and the management options include conservative treatment, percutaneous pinning, IOW, screw fixation, plate fixation, and external fixation. Closed reduction and percutaneous pinning are most frequently used for dislocated or unstable proximal phalangeal fractures, and good outcomes have been reported. Eberlin et al. reported a TAM of ≥ 250° in 80% of the 41 cases [6]. However, for shaft fractures involving more than three fragments, fixation with percutaneous pinning or screws alone is challenging, and plate fixation or IOW is considered a more suitable option. Plate fixation provides good stability but some authors reported to cause a high rate of complications and poor treatment outcomes. Pun et al. reported that the outcomes for 36 cases of fractures of the proximal phalanges treated with either AO miniplate or screw fixation were good in 27.8%, fair in 36.1%, and poor in 36.1% cases [7]. Kurzen et al. reported complications in 34 out of 55 (62%) patients with proximal phalanx fractures treated plate fixation, including incomplete bone healing, plate loosening, infection, and CRPS [8]. In addition, adhesions between plates and the extensor tendon are often seen, resulting in a limited joint ROM, and good treatment outcomes were challenging to obtain. Onishi et al. compared plate and screw fixations in open reduction and internal fixation surgery for proximal phalangeal fractures and reported that dorsal plate fixation resulted in limited ROM caused by adhesions of the extensor tendons on the implant [9]. On the other hand, good outcomes can be obtained in plate and screw fixation if the implant and tendon adhesions do not occur. Guang et al. reported the mean TAM of 234.60 ± 22.63°was obtained by placing the plate on dorsolateral side of proximal phalanx through the dorsolateral approach [10]. Sahin et al. reported the mean TAM of 259°. To prevent postoperative adhesion of the extensor tendons, it is necessary to repair the periosteum after placing the implant so that the implant and the extensor tendon do not directly come into contact [11].

Because our method uses small-diameter Kirschner wires and stainless steel wires, with which we can repair the periosteum after implant placement, the implant does not directly touch the extensor tendon.

There have only been a few reports of IOW, including tension band wiring, which included all simple fracture cases. Pehlivan et al. performed tension band wiring for 12 cases of closed unstable diaphyseal/base transverse fractures and reported a mean TAM of 92% and excellent outcome in all cases [12]. Although these reports did not include a detailed description of whether the periosteum was repaired after implant placement or not, the favorable outcomes could be due to less frequent adhesions of the extensor tendon and the implant compared with those in plate fixation cases. Teoh et al. reported that they performed firm fixation using cerclage wiring and miniplates for multi-fragment fractures of the metacarpal, proximal, and middle phalanges and sutured the periosteum when possible [13]. The outcome of five cases of proximal phalangeal fractures treated with this method was good with a mean TAM of 224°.

Firm fixation and early rehabilitation are recommended to prevent postoperative extensor tendon adhesions and ROM limitation [14,15], Miller et al. reported that functional recovery (ROM, pain relief, return to work, and grip strength) was mostly achieved within the first 6 postoperative weeks [15]. Al-Qattan and Al-Zahrani reported that of the 15 patients with long oblique/spiral fractures of the phalangeal shaft who underwent open cerclage wire fixation, 12 achieved full-range motion (TAM 260°) by starting ROM training immediately after surgery. They reported that cerclage wire fixation was firm enough for early ROM exercise, resulting in better treatment outcomes [16]. When using IOW, proper repositioning can be obtained as bone fragments are reduced one by one; moreover, firm stability can be obtained by compressing bone fragments, allowing early exercise therapy under Burkhalter fixation. For the DIP/PIP joints, we started ROM training immediately after surgery to prevent joint contractures. For the MP joints, we used flexion fixation to prevent extension contractures. Even when the PIP joints are left free, flexion contractures can occur. Therefore, we instructed the patients to regularly perform PIP joint extension exercises.

This study has some limitations. First, two of the five cases had a postoperative follow-up period of <1 year (8 and 10 months). However, good TAM and %TAM was obtained in both cases within 3 months after surgery, which assures good clinical outcomes even if the follow-up period is short. Second, the number of cases is small. Therefore, a large sample size is required to confirm the observations in the present study. A second limitation is that no patient reported outcomes like the MHQ are used.

In conclusion, the present study describes cases of proximal phalangeal fractures involving more than three fragments that were challenging to treat with percutaneous pinning or screw fixation and were treated with IOW. Plate and screw can be used as an osteosynthesis in suitable cases. However, although IOW certainly requires proficiency in the procedure, it is possible to fix small bone fragments that are difficult to fix with plate and screw, and so I think that it is a good option for fragmented proximal phalangeal fractures. The treatment outcomes were positive without deformity or the patient complaining of pain, with a mean TAM of 237° and mean %TAM of 94%; all five cases were assessed as having excellent outcomes, based on the ASSH criteria. A good range of TAM was achieved by starting ROM training for the DIP and PIP joints immediately after surgery and fixing the MP joints in the flexion position.
